# Circulating palmitoyl sphingomyelin levels predict the 10-year increased risk of cardiovascular disease death in Chinese adults: findings from the Da Qing Diabetes Study

**DOI:** 10.1186/s12933-023-02116-8

**Published:** 2024-01-20

**Authors:** Xin Qian, Hongmei Jia, Jinping Wang, Siyao He, Meng Yu, Xinxing Feng, Qiuhong Gong, Yali An, Xuan Wang, Na Shi, Hui Li, Zhongmei Zou, Guangwei Li, Yanyan Chen

**Affiliations:** 1 Endocrinology Centre, Fuwai Hospital, Chinese Academy of Medical Sciences and Peking Union Medical College, Beijing, China; 2 Institute of Medicinal Plant Development, Chinese Academy of Medical Sciences and Peking Union Medical College, Beijing, China; 3 Department of Cardiology, Da Qing First Hospital, Da Qing, China; 4 Department of Endocrinology, China-Japan Friendship Hospital, Beijing, China

**Keywords:** All-cause death, CVD death, Predictor, PSM

## Abstract

**Background:**

Higher levels of palmitoyl sphingomyelin (PSM, synonymous with sphingomyelin 16:0) are associated with an increased risk of cardiovascular disease (CVD) in people with diabetes. Whether circulating PSM levels can practically predict the long-term risk of CVD and all-cause death remains unclear. This study aimed to investigate whether circulating PSM is a real predictor of CVD death in Chinese adults with or without diabetes.

**Methods:**

A total of 286 and 219 individuals with and without diabetes, respectively, from the original Da Qing Diabetes Study were enrolled. Blood samples collected in 2009 were used as a baseline to assess circulating PSM levels. The outcomes of CVD and all-cause death were followed up from 2009 to 2020, and 178 participants died, including 87 deaths due to CVD. Cox proportional hazards regression was used to estimate HRs and their 95% CIs for the outcomes.

**Results:**

Fractional polynomial regression analysis showed a linear association between baseline circulating PSM concentration (log-2 transformed) and the risk of all-cause and CVD death (p < 0.001), but not non-CVD death (p > 0.05), in all participants after adjustment for confounders. When the participants were stratified by PSM-tertile, the highest tertile, regardless of diabetes, had a higher incidence of CVD death (41.5 vs. 14.7 and 22.2 vs. 2.9 per 1000 person-years in patients with and without diabetes, respectively, all log-rank p < 0.01). Individuals with diabetes in the highest tertile group had a higher risk of CVD death than those in the lowest tertile (HR = 2.73; 95%CI, 1.20–6.22).

**Conclusions:**

Elevated PSM levels are significantly associated with a higher 10-year risk of CVD death, but not non-CVD death, in Chinese adults with diabetes. These findings suggest that PSM is a potentially useful long-term predictor of CVD death in individuals with diabetes.

**Supplementary Information:**

The online version contains supplementary material available at 10.1186/s12933-023-02116-8.

## Highlights

Why did we undertake this study?

- Diabetes is associated with a higher risk of all-cause and cardiovascular disease (CVD) deaths after control of blood pressure, glucose, and lipid.
- Circulating palmitoyl sphingomyelin (PSM) level is associated with an increased risk of CVD in Chinese patients with diabetes.

What is the specific question we wanted to answer?

- Is PSM a practical predictor for CVD death?

What did we find?

- Circulating PSM levels steadily predicted a 10-year increased risk of CVD death after adjusted for SBP, HbA_1c_, LDL-c, creatinine, prevalent CVD, and the use of alcohol and statins but not non-CVD death, in individuals with diabetes.

What are the implications of our findings?

- PSM determination may be useful for predicting the long-term risk of CVD death.

**Supplementary Information:**

The online version contains supplementary material available at 10.1186/s12933-023-02116-8.

## Background

The number of people with diabetes and cardiovascular diseases (CVDs) is predicted to increase [1, 2]. Most diabetes-related morbidity and excess mortality is associated with vascular complications, even after control of blood pressure, glucose, and lipid. Diabetes studies therefore aim to prevent and reduce CVD and to identify people at risk early, especially those at residual high risk. Molecular lipidomics analyses of lipid metabolite composition and biochemical pathways are particularly important because of the role of lipids in CVD pathophysiology. It is possible to predict disease states more accurately using the metabolome, which provides biochemical feedback across all omics layers.

As a component of plasma membranes and membrane microdomains, such as caveolae, lipid rafts, and clathrin-coated pits, sphingomyelin (SM) plays a critical role in transmembrane signaling and atherosclerosis [3, 4]. SM levels are associated with the risk of CVD and total death [5] and incident heart failure [6]. In addition, the association between SM levels and death differs according to the length of the acylated saturated fatty acids in participants aged > 65 years in the Cardiovascular Health Study [7]. In addition to its association with CVDs, lipid metabolism has also been associated with an increased risk of developing diabetes and diabetes-related complications. Several circulating metabolites associated with type 2 diabetes risk in relatively lean Chinese adults [8]. Compared with healthy controls, the serum levels of SM were significantly lower in patients with pre-diabetes and diabetes [9]. However, the current evidence on the role of SM in the development of diabetes-related CVD is limited. Circulating phospholipid species are more pronounced lower in individuals with diabetes with CVD than that in those without CVD [10]. Increased plasma SM levels may be associated with the benefits of empagliflozin in patients with type 2 diabetes mellitus at risk of CVD [11]. Adults with type 2 diabetes with SM containing a very-long-chain saturated fatty acid have a lower CVD risk [12].

In contrast, higher levels of palmitoyl SM (PSM, synonymous with SM [16:0]) are associated with an increased risk of CVD in individuals with diabetes through untargeted metabolomics analysis in a discovery dataset and targeted analysis in a verified dataset [13]. However, it remains unclear whether circulating PSM levels can practically predict the long-term risk of CVD and all-cause mortality. In this study, based on the Da Qing Diabetes Study cohort and its long-term follow-up data, we aimed to investigate the predictive effect of the plasma levels of PSM on the 10-year risk of death among Chinese adults aged 45–86 years.

## Methods

### Study design and participant enrolment

All the participants in this study were from a prospective cohort of the Da Qing Diabetes Study. Detailed enrolment has been reported previously [14]. Briefly, in 1985, 3956 individuals with 2-h plasma glucose level ≥ 6.7 mmol/L after standard breakfast accepted a 75-g standard oral glucose tolerance test (OGTT). Among these individuals, 630 were newly diagnosed with diabetes according to the WHO criteria 1985 for type 2 diabetes [15], and 519 were identified as having normal glucose tolerance. In a follow-up study in 2009, 505 participants (286 with diabetes and 219 without diabetes) with plasma PSM levels were included in this study (Additional file 1: Fig. S1), and the data were used as the baseline. The clinical outcomes of all-cause, CVD, and non-CVD deaths were traced up to 31 December 2020.

### Data collection and outcome assessment

Medical records and death certificates were reviewed to verify the data collected from proxy informants using standardized questionnaires. Data collection has been described previously [14, 16, 17]. In brief, all participants were from the Da Qing Diabetes Study, the baseline information of age, sex, smoking status, and the use of alcohol were collected through face-to-face interview in 2009. The data of height, weight, and blood pressure were collected by physical examination, the level of HbA_1c_, lipid, and creatinine were measured in Da Qing First Hospital based on a standard protocol. Diabetes status was assessed through OGTT results for non-diabetic individuals and through medical record review, laboratory examination, and records of receiving glucose-lowering medications. CVD events were defined as non-fatal or fatal myocardial infarction or sudden death, hospital admission for heart failure, or non-fatal or fatal stroke. CVD deaths were defined as death due to myocardial infarction, sudden death, heart failure, or stroke. The information of all-cause death was first collected by proxy informants using standardized questionnaires, then confirmed by medical records review and/or death certificate. Causes of death were determined from review of medical records and death certificates. The CVD death also confirmed by medical records (including the data of clinical symptoms, electrocardiogram, coronary revascularization, enzyme, or X‐ray examinations) and/or death certificate if available. The earliest date of outcome recognition was defined as onset. Outcomes were independently evaluated by two physicians, and a third senior physician resolved disagreements. This study was approved by the Ethics Committee of the Fuwai Hospital. Written informed consent was obtained from all participants or representatives of deceased participants.

### Palmitoyl sphingomyelin level measurement

Fasting EDTA plasma samples were used to measure PSM levels, as reported in previous studies[6, 7, 18] because EDTA inhibits sphingomyelinases and ensures stable PSM levels. All samples were collected in 2009, immediately separated, frozen at − 80 °C and thawed for analysis. Ultra-high-performance liquid chromatography-tandem mass spectrometry (UPLC-MS/MS) analysis was used to determine plasma PSM concentrations. UPLC-MS/MS analysis was performed on a Waters ACQUITY Ultra Performance LC System (Waters Corporation, Milford, MA) equipped with a BEH C18 column (100 mm × 2.1 mm, 1.7 µm). Quantitative analysis was performed using a chromatographic reference compound, PSM (Y0852; CAS 6254-89-3; A.V.T. Pharmaceutical Co., Ltd.).

For hierarchical analysis, the participants were stratified into three groups based on the PSM level tertile: Group 1, PSM ≤ 7.51 µg/ml; Group 2, PSM = 7.51–11.05 µg/ml; and Group 3, PSM > 11.05 µg/ml. PSM levels were ≤ 8.78, 8.78–12.61, and > 12.61 µg/ml in Groups 1, 2, and 3, respectively, in individuals with diabetes. In those without diabetes, the corresponding PSM levels were ≤ 6.85, 6.85–9.21, and > 9.21 µg/ml in Groups 1, 2, and 3, respectively.

### Statistical analyses

For continuous variables, univariate analysis was used to determine the distribution. Normally distributed continuous variables are presented as the mean ± standard deviation and compared among the groups using a *t-test*. Right-skewed distributed variables are reported as median (interquartile range, 25–75th percentiles) and compared using the Wilcoxon rank-sum test. Categorical variables are presented as numbers and percentages (%) and analyzed using chi-squared or Fisher’s exact tests, as appropriate. PSM was analyzed as continuous (log-2 transformed) and grouped (tertiles) variables. The homoeostatic model assessment for insulin resistance (HOMA_IR) was calculated using the following formula: HOMA_IR = (fasting glucose [mmol/L] × fasting insulin [mU/L]/22.5) [19].

Log-minus-log plots were used to assess the proportional hazards assumption. The time-to-event survival curve for death was created using the cumulative incidence method and compared among groups using log-rank tests. Cox proportional hazards regression was used to estimate hazard ratios (HRs) and 95% confidence intervals (CIs) for each outcome, with adjustment for confounders, including age, sex, smoking status, systolic blood pressure (SBP), hemoglobin A1c (HbA_1c_) level, and low-density lipoprotein cholesterol (LDL-c) level, creatinine level, prevalent CVD, and the use of alcohol and statins. A fractional polynomial regression model was used to evaluate the log-linear association between PSM levels and death, with adjustment for the covariables. Cox regression models were used to calculate HRs [20]. To compare the influence of PSM levels on death with the traditional risk factor, age increased per 10 years and SBP increased by 10 mmHg were used in the Cox model. Missing data points (< 0.01% of the data) were mean-substituted [21].

All statistical analyses were performed using SAS version 9.4 (SAS, Cary, NC, USA) and Stata SE version 16.0 (StataCorp) software. Two-sided p < 0.05 was considered statistically significant.

## Results

### Baseline characteristics

The basic clinical characteristics of the study participants are shown in Table 1. A total of 505 participants were included in this study. The average age was > 60 years (64.9 ± 7.6 years) in all participants, of whom 222 (44%) were male and 175 (35%) were smokers. The average duration of diabetes was 18.5 years (18.5 ± 8.3 years) in individuals with diabetes. The plasma levels of PSM in individuals with diabetes were higher than that in those without diabetes (10.34 [7.51–24.08] vs. 8.03 [6.07–10.14] µg/ml, p < 0.001). Participants with diabetes had higher SBP and triglyceride levels (147.9 ± 22.5 vs. 138.1 ± 20.0 mmHg and 2.0 ± 1.4 vs. 1.8 ± 1.0 mmol/L, respectively; all p < 0.05) and higher insulin resistance than those without diabetes (HOMA_IR, 3.13 [1.80–7.23] vs. 1.24 [1.02–2.20]; p < 0.01). Diabetes group also had higher creatinine level (66.4 [54.7–80.5] vs. 61.5 [53.2–75.9] µmol/L, p = 0.023) and higher frequency of prevalent CVD (79 [27.6%] vs. 43 [19.6%], p = 0.038) than non-diabetes group.

**Table 1:** Clinical characteristics at baseline and outcomes over 10 years (2009–2020)

	All participants	Individuals without diabetes	Individuals with diabetes	P value
n	505	219	286	
*At baseline (2009)*				
Age, years	64.9 ± 7.6	64.2 ± 8.0	65.5 ± 7.3	0.071
Male, n (%)	222 (44)	103 (47)	119 (42)	0.240
Smoking, n (%)	175 (35)	85 (39)	90 (31)	0.090
BMI, kg/m^2^	25.5 ± 3.6	25.4 ± 3.7	25.6 ± 3.5	0.536
SBP, mmHg	143.5 ± 22.0	138.1 ± 20.0	147.9 ± 22.5	< 0.001
HbA_1c_, %	7.1 ± 1.6	5.9 ± 0.5	8.0 ± 1.7	< 0.001
TG, mmol/L	1.9 ± 1.2	1.8 ± 1.0	2.0 ± 1.4	0.013
LDL-c, mmol/L	3.0 ± 0.9	3.0 ± 0.8	3.1 ± 1.0	0.167
Uric acid, mmol/L	318.9 ± 87.8	323.2 ± 85.9	315.3 ± 89.4	0.319
Creatinine, µmol/L	64.6 (54.1–77.1)	61.5 (53.2–75.9)	66.4 (54.7–80.5)	0.023
HOMA_IR	2.11 (1.18–4.52)	1.24 (1.02–2.20)	3.13 (1.80–7.23)	< 0.001
DM duration, years	–	–	18.5 ± 8.3	–
Prevalent CVD, n (%)	122 (24.2)	43 (19.6)	79 (27.6)	0.038
Alcohol use, n (%)	79 (15.6)	44 (20.0)	35 (12.2)	0.017
PSM, µg/ml	9.04 (6.69–13.22)	8.03 (6.07–10.14)	10.34 (7.51–24.08)	< 0.001
*Follow-up (2009–2020)*				
Anti-hypertensives, n (%)	331 (65.5)	123 (56.2)	208 (72.7)	< 0.001
Lipid-lowering agents, n (%)	268 (53.1)	97 (44.3)	171 (59.8)	0.001
Statins, n (%)	218 (43.2)	81 (37.0)	137 (47.9)	0.014
All-cause death, n (%)	178 (35.2)	49 (22.4)	129 (45.1)	< 0.001
CVD death, n (%)	87 (17.2)	20 (9.1)	67 (23.4)	< 0.001
Non-CVD death, n (%)	91 (18.0)	29 (13.2)	62 (21.7)	0.015

Data are presented as mean ± standard deviation or median (interquartile range, 25th–75th percentiles), except for qualitative variables, which are expressed as n (%). *CVD* cardiovascular disease, *SBP* systolic blood pressure, *HbA*_*1c*_ glycated hemoglobin, *TG* triglyceride, *LDL-c* low-density lipoprotein cholesterol, *DM* diabetes mellitus, *PSM* palmitoyl sphingomyelin

### Outcomes

A total of 178 (35.2%) patients had all-cause death during follow-up, of whom 87 (48.9%) died of CVD (Table 1 and Additional file 1: Fig. S1). Patients with diabetes had higher death rates, including all-cause, CVD, and non-CVD death, than those without diabetes (all p < 0.05).

### PSM levels associated with outcomes

Multivariable fractional polynomial (MFP) regression analysis showed a linear association between the risk of each outcome (log HR) and circulating PSM concentration (log-2 transformed) (Additional file 1: Fig. S2). In all participants, a progressively higher risks of all-cause and CVD death was associated with PSM levels after adjustment for confounders (p < 0.05), but not non-CVD death. In the subgroups, the linear association between PSM levels and all-cause and CVD death persisted in diabetes group (p < 0.05, Additional file 1: Fig. S2d and e). The linear association between PSM levels and non-CVD death was not significant (p > 0.05, Additional file 1: Fig. S2c, f and i).

### PSM levels predicted the risks of all-cause and CVD death

Table 2 shows the association between circulating PSM levels (log-2 transformed) and the outcomes. In all participants, per doubling in PSM (µg/ml) level was associated with all-cause death (HR = 1.37; 95% CI, 1.12–1.68; p = 0.002) after adjustment for age, sex, smoking status, HbA_1c_ level, SBP, LDL-c level, creatinine level, prevalent CVD, and the use of statins and alcohol. The risk of CVD death increased by 50% (HR = 1.50; 95% CI, 1.12–2.00; p = 0.006) per twofold increase in PSM (µg/ml) levels. In patients with diabetes, the risks of all-cause and CVD death increased by 47% and 53%, respectively, per twofold increase in PSM (µg/ml) level (p < 0.05). The HRs were 1.18 (95% CI, 0.73–1.92) for all-cause death and 1.31 (95% CI, 0.65–2.63) for CVD death in individuals without diabetes. PSM levels were not associated with the risk of non-CVD death in either the diabetes or non-diabetes group (HR = 1.39; 95% CI, 0.98–1.96; p = 0.06 and HR = 1.01; 95% CI, 0.51–2.00; p = 0.98, respectively), consistent with the results of the MFP regression analysis.

**Table 2:** The association between circulating PSM (log-2 transformed) levels and outcomes

	All participants (n = 505)	P value	Individuals with diabetes (n = 286)	p value	Individuals without diabetes (n = 219)	p value
All-cause death, n (%)	178 (35.2)		129 (45.1)		49 (22.4)	
Crude HR (95% CI)	1.69 (1.48–1.93)	< 0.001	1.52 (1.30–1.76)	< 0.001	1.82 (1.34–2.47)	< 0.001
Model 1 HR (95% CI)	1.61 (1.41–1.83)	< 0.001	1.46 (1.25–1.69)	< 0.001	1.86 (1.34–4.51)	< 0.001
Model 2 HR (95% CI)	1.47 (1.28–1.73)	< 0.001	1.42 (1.21–1.67)	< 0.001	1.67 (1.17–2.36)	0.004
Model 3 HR (95% CI)	1.37 (1.12–1.68)	0.002	1.47 (1.16–1.87)	0.002	1.18 (0.73–1.92)	0.50
CVD death, n (%)	87 (17.2)		67 (23.4)		20 (9.1)	
Crude HR (95% CI)	2.04 (1.69–2.45)	< 0.001	1.73 (1.40–2.13)	< 0.001	2.73 (1.74–4.28)	< 0.001
Model 1 HR (95% CI)	1.91 (1.58–2.30)	< 0.001	1.63 (1.32–2.01)	< 0.001	2.82 (1.75–4.56)	< 0.001
Model 2 HR (95% CI)	1.75 (1.43–2.14)	< 0.001	1.58 (1.27–1.98)	< 0.001	2.31 (1.39–3.83)	0.001
Model 3 HR (95% CI)	1.50 (1.12–2.00)	0.006	1.53 (1.10–2.14)	0.012	1.31 (0.65–2.63)	0.44
Non-CVD death, n (%)	91 (18.0)		62 (21.7)		29 (13.2)	
Crude HR (95% CI)	1.38 (1.13–1.68)	0.001	1.30 (1.04–1.62)	0.023	1.30 (0.83–2.01)	0.25
Model 1 HR (95% CI)	1.33 (1.09–1.61)	0.004	1.26 (1.01–1.58)	0.04	1.29 (0.81–2.06)	0.29
Model 2 HR (95% CI)	1.23 (1.00–1.51)	0.046	1.24 (0.98–1.57)	0.069	1.22 (0.73–2.02)	0.45
Model 3 HR (95% CI)	1.24 (0.93–1.66)	0.15	1.39 (0.98–1.96)	0.062	1.01 (0.51–2.00)	0.98

Model 1: Adjusted for age (per 10 years) and sex; Model 2: plus adjusting for smoking status, SBP (per 10 mmHg), HbA_1c_ level, and LDL-c level; Model 3: plus adjusting for creatinine level, prevalent CVD, and the use of statins and alcohol

*PSM* palmitoyl sphingomyelin, *CVD* cardiovascular disease, *HR* hazard ratio, *CI* confidence interval, *HbA*_*1c*_ glycated hemoglobin, *SBP* systolic blood pressure, *LDL-c* low-density lipoprotein cholesterol

To compare the cumulative incidence of CVD death, participants were grouped according to the tertile of PSM levels (Table 3). In all participants, PSM levels were 5.98 (5.02–6.68), 9.06 (8.35–10.04), and 25.74 (13.41–36.83) µg/ml in Groups 1, 2, and 3, respectively. The difference in PSM concentration between Group1 and 3 differed according to diabetes status. In participants with diabetes, the PSM levels were more than 5 times higher in Group3 than in Group1 (33.85 [24.08–44.03] vs. 6.23 [5.11–7.53] µg/ml), whereas in those without diabetes, it was less than 3 times (14.02 [10.14–25.33] vs. 5.23 [4.39–6.07] µg/ml). In individuals with diabetes, the incidence rate of CVD death in Group 3 was significantly higher than that in Group 1 (41.5 vs. 14.7 per 1000 person-years, p < 0.001). The same trend of difference in the incidence rate of CVD death was observed in individuals without diabetes (22.2 vs. 2.9 per 1000 person-year, p < 0.001).

**Table 3:** Incidence of cardiovascular disease death among tertile groups with different circulating PSM levels

	Group 1	Group 2	Group 3	P value
All participants, n	170	168	167	
PSM, µg/ml	5.98 (5.02–6.68)	9.06 (8.35–10.04)	25.74 (13.41–36.83)	< 0.001
Incidence per 1000 person-years (95% CI)	7.8 (4.6–13.2)	13.7 (9.0–21.1)	38.7 (29.5–50.8)	< 0.001
Individuals with diabetes, n	95	95	96	
PSM, µg/ml	6.23 (5.11–7.53)	10.35 (9.56–11.11)	33.85 (24.08–44.03)	< 0.001
Incidence per 1000 person-years (95% CI)	14.7 (8.8–24.0)	24.8 (16.5–36.3)	41.5 (31.6–53.2)	< 0.001
Individuals without diabetes, n	73	73	73	
PSM, µg/ml	5.23 (4.39–6.07)	8.03 (7.29–8.68)	14.02 (10.14–25.33)	< 0.001
Incidence per 1000 person-years (95% CI)	2.9 (0.7–11.0)	4.2 (1.4–12.4)	22.2 (14.0–34.2)	< 0.001

*PSM* palmitoyl sphingomyelin, *CI* confidence interval

**Fig. 1:**
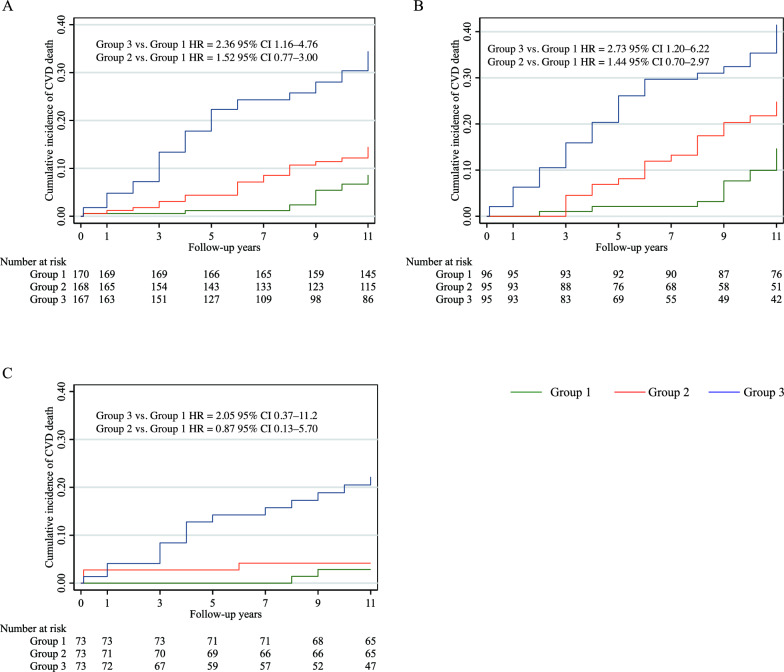
Kaplan–Meier failure estimate according to tertile group of PSM cardiovascular disease death in **A** all participants, **B** individuals with diabetes, and **C** individuals without diabetes. *HR* hazard ratio, *CI* confidence interval. HR calculated after adjustment for age, sex, smoking status, SBP, HbA_1c_ level, LDL-c level, creatinine level, prevalent CVD, and the use of statins and alcohol

Figure 1 shows the Kaplan–Meier failure estimate according to the tertile of PSM levels. In all participants, the Kaplan–Meier curves of the tertile groups separated after 1 year of follow-up, and the risk of CVD death was twofold higher in Group 3 than in Group 1 (Fig. 1A). In participants with diabetes, the Kaplan–Meier curves of the tertile groups were separated from the 4-year follow-up (log-rank p < 0.01). The risk of CVD death in Group 3 was more than 2 times higher than that in Group 1 after adjustment for traditional risk factors (HR = 2.73; 95% CI, 1.02–6.22; p = 0.017; Fig. 1B). In individuals without diabetes, the cumulative incidence rate of CVD death in Group 3 was higher than that in Group 1 (22.2% vs. 2.9%, log-rank p < 0.001, Fig. 1C). The HR of CVD death in Group 3 had a relatively broad 95% CI, possibly owing to the limited number of deaths.

## Discussion

A key finding from this prospective study was the predictive effect of circulating levels of PSM on subsequent decade death. The predictive value of PSM for CVD death is more prominent in individuals with diabetes than in those without diabetes because they have significantly higher PSM levels than those without diabetes. Through fractional polynomial Cox analysis (adjusted for age, sex, smoking status, HbA_1c_ level, SBP, LDL-c level, creatinine level, and the use of statins and alcohol), PSM levels were found to be linearly associated with the risks of all-cause and CVD death in all participants and those with diabetes. In multivariable Cox model analysis, per twofold increase in PSM (µg/ml) level was associated with 50% and 37% high risk of CVD and all-cause death, respectively. In the subgroup analysis, the association was persistent in individuals with diabetes. Similarly, the 11-year follow-up data showed that the cumulative incidence rate of CVD death was higher in individuals in the highest PSM tertile than in the lowest, both with and without diabetes. Interestingly, the risk of non-CVD death was not significantly associated with higher PSM levels.

Even after taking medication for hypertension, dyslipidaemia and diabetes, the risk of CVD and all-cause death was still higher in people with diabetes than in people without diabetes. In addition, some people are at a higher risk than others, even though all of them have diabetes. Therefore, our study focused on identifying those at higher risk of developing long-term outcomes at an early stage. We previously reported that an increased level of PSM is associated with a high risk of CVD in patients with type 2 diabetes through a cross-sectional data analysis [13]. First, using a non-targeted metabolomics method, we found that changes in metabolites, including lipid and fatty acid contents, were significantly associated with cardiovascular risk in patients with diabetes. After targeted analysis, a higher level of PSM was associated with a higher risk of CVD in individuals with diabetes. Then, we validated this finding in a separate population and have established a relationship between PSM levels and CVD risk with concrete information.

In this study, we firstly assessed the predictive effect of PSM levels on the risk of CVD death over a decade follow-up. People with diabetes and higher level of PSM have a higher risk of CVD and all-cause death. PSM is considered one of the most prevalent species of SM [22]. Plasma SM levels are positively and independently associated with coronary artery disease in a cross-sectional study [23] and can predict myocardial infarction and cardiovascular death in patients with acute coronary syndrome [24]. Our results are consistent with the results of clinic-based studies of the association between plasma SM levels and CVD. Notably, in the present study, the association between plasma PSM levels and CVD death persisted even after adjustment for LDL-c levels and statins, suggesting the strong predictive power of PSM for CVD death in people with diabetes, especially for residual high risk after control of blood pressure, blood glucose, and lipids. Mihyun Bae et al., reported that SM levels differ by alcohol use status for the SM can be hydrolyzed to ceramide by sphingomyelinase under stress [25]. Patients with chronic kidney disease had higher levels of plasma Ceramide (d18:1/16:0) [26]. Additionally, higher plasma SM (16:0) levels mediated the association between reduced eGFR and incident heart failure [18] and were significantly associated with greater albuminuria [27]. Therefore, in the present analysis, creatinine level and alcohol use were adjusted, and the significant association between higher levels of PSM and CVD death remained.

However, the function of SM differs from the different carbon chains and species and the results were inconsistent. Some studies showed the association between higher plasma SM-16 levels and increased risk of heart failure and higher SM-20, SM-22, and SM-24 levels and decreased risk of heart failure [6]. SM (16:0) was vital for predicting CVD death in a case-cohort subset from the Action in Diabetes and Vascular Disease: Preterax and Diamicron-MR Controlled Evaluation trial [28], whereas another study demonstrated that SM (16:0, 16:1) was associated with lower cardiac vagal tone in individuals recently diagnosed with type 2 diabetes [29]. In the present study, we provide clear evidence of the predictive effect of PSM levels containing palmitic acid (16:0) on death, particularly CVD death. These findings may help to identify people at increased risk of long-term outcomes a decade earlier. SM can be synthesized from ceramide by synthase and converted back to ceramide by sphingomyelinase [3] preserving the acylated palmitic acid. Therefore, the direct cardiotoxic effects of sphingolipids is worthy of attention. Foundational basic studies through transgenic mouse models, such as mice overexpressing long-chain acyl- CoA synthetase in the heart [30] and cardiac-specific glycosylphosphatidylinositol-anchored human lipoprotein lipase [31], have demonstrated that ceramide accumulation in cardiac myocytes significantly contributes to the development of cardiomyopathy.

We also found that PSM, which contained palmitic acid (16:0), was correlated with HbA_1c_ level and HOMA_IR in individuals with diabetes. These results are consistent with those of other studies on the association between SM or fatty acid levels and insulin resistance. Higher levels of SM (carrying 18:0, 20:0, 22:0, or 24:0) were associated with lower fasting insulin levels, and the homoeostatic model assessed HOMA_IR and HOMA-B among individuals with normal BMI in the Strong Heart Family Study cohort study [32]. However, the reduction of SM levels in the plasma membranes of liver cells leads to an improvement in tissue and whole-body insulin sensitivity in *Sptlc2* and *sms2* knockout mice [33]. Palmitic acid (16:0) (but not stearic acid [18:0]) induces impaired ß-cell function and insulin resistance [34], which may be caused by abnormal lipid distribution, endoplasmic reticulum expansion, and stress [35]. The elevated hepatic PSM content promotes inflammation by altering membrane lipid composition and toll-like receptor-4 signaling [36]. Therefore, another possible explanation for the association between PSM levels and CVD death lies in the dysfunction of ß-cells and insulin sensitivity and inflammation, which are pivotal patterns in the development of diabetes-related vascular complications, such as atherosclerosis. Changes in plasma PSM levels seem to represent a change in systemic inflammation and insulin resistance level, and this change may be an early reaction of the body to stress, although the different effects of PSM and SM levels remain unknown.

This study has several strengths. First, the participants were from a long-term cohort in the Da Qing Diabetes Study. Some follow-up data have been reported, and 94% of the participants were assessed for outcomes [16]. Second, the average age of the participants at recruitment was > 60 years, with a high frequency of outcomes. Third, the association between PSM levels and CV events was identified by untargeted metabolomics, quantitatively confirmed by targeted metabolomics, and validated with external data. However, the relatively small sample size and single ethnicity of the participants limited speculation to other individuals. Alcohol use is complicated in China for the use of liquor and rice wine. So, we can only define the alcohol use as yes or no in the Cox model. The underlying mechanism of the predictive effect of higher PSM levels on the increased risk of CVD death requires further studies.

## Conclusions

This prospective study found that a higher level of PSM is associated with a subsequent 10-year higher all-cause death, especially CVD death, in older individuals with diabetes. These findings suggest that PSM is a potentially useful long-term predictor of CVD death. Large-scale longitudinal studies are needed to determine the usefulness of this metabolite as a predictor of diabetes-related complications.

### Supplementary Information

**Additional file 1: Fig. S1.** Study flowchart. IGT, impaired glucose tolerance; NGT, normal glucose tolerance; PSM, palmitoyl sphingomyelin; CVD, cardiovascular disease. **Fig. S2.** Fractional polynomial regression. a) All-cause death, b) CVD death, and c) non-CVD death in all participants; d) all-cause death, e) CVD death, and f) non-CVD death in individuals with diabetes; g) all-cause death, h) CVD death, and i) non-CVD death in individuals without diabetes. PSM, palmitoyl sphingomyelin; CVD, cardiovascular diseases. HR calculated after adjustment for age, sex, smoking status, SBP, HbA_1c_, LDL-c level, creatinine level, prevalent CVD, and the use of statins and alcohol.

## Data Availability

The datasets used and/or analyzed during the current study are available from the corresponding author upon reasonable request.

## Abbreviations

PSM: Palmitoyl sphingomyelin
SM: Sphingomyelin
CVD: Cardiovascular disease
HRs: Hazard ratios
CIs: Confidence intervals
UPLC-MS/MS: Ultra-high-performance liquid chromatography–tandem mass spectrometry
HOMA_IR: Homoeostatic model assessment for insulin resistance
SBP: Systolic blood pressure
HbA_1c_: Hemoglobin A1c
LDL-c: Low-density lipoprotein cholesterol
MFP: Multivariable fractional polynomial

## References

[1] International Diabetes Federation. Diabetes and cardiovascular disease. Brussels, Belgium: International Diabetes Federation; 2016. www.idf.org/cvd.

[2] Yang JJ, Yu D, Wen W, Saito E, Rahman S, Shu XO, Chen Y, Gupta PC, Gu D, Tsugane S (2019). Association of diabetes with all-cause and cause-specific mortality in Asia: a pooled analysis of more than 1 million participants. JAMA Netw Open.

[3] Bienias K, Fiedorowicz A, Sadowska A, Prokopiuk S, Car H (2016). Regulation of sphingomyelin metabolism. Pharmacol Rep.

[4] Barenholz Y (2004). Sphingomyelin and cholesterol: from membrane biophysics and rafts to potential medical applications. Subcell Biochem.

[5] Sigruener A, Kleber ME, Heimerl S, Liebisch G, Schmitz G, Maerz W (2014). Glycerophospholipid and sphingolipid species and mortality: the Ludwigshafen Risk and Cardiovascular Health (LURIC) study. PLoS ONE.

[6] Lemaitre RN, Jensen PN, Hoofnagle A, McKnight B, Fretts AM, King IB, Siscovick DS, Psaty BM, Heckbert SR, Mozaffarian D (2019). Plasma ceramides and sphingomyelins in relation to heart failure risk. Circ Heart Fail.

[7] Fretts AM, Jensen PN, Hoofnagle AN, McKnight B, Sitlani CM, Siscovick DS, King IB, Psaty BM, Sotoodehnia N, Lemaitre RN (2021). Circulating ceramides and sphingomyelins and risk of mortality: the cardiovascular health study. Clin Chem.

[8] Bragg F, Kartsonaki C, Guo Y, Holmes M, Du H, Yu C, Pei P, Yang L, Jin D, Chen Y (2022). Circulating metabolites and the development of type 2 diabetes in Chinese adults. Diabetes Care.

[9] Sui J, He M, Wang Y, Zhao X, He Y, Shi B (2019). Sphingolipid metabolism in type 2 diabetes and associated cardiovascular complications. Exp Ther Med.

[10] Garcia-Fontana B, Morales-Santana S, Diaz Navarro C, Rozas-Moreno P, Genilloud O, Vicente Perez F, Perez del Palacio J, Munoz-Torres M (2016). Metabolomic profile related to cardiovascular disease in patients with type 2 diabetes mellitus: A pilot study. Talanta.

[11] Deng X, Zhang C, Wang P, Wei W, Shi X, Wang P, Yang J, Wang L, Tang S, Fang Y (2022). Cardiovascular benefits of empagliflozin are associated with gut microbiota and plasma metabolites in type 2 diabetes. J Clin Endocrinol Metab.

[12] Jensen PN, Fretts AM, Hoofnagle AN, McKnight B, Howard BV, Umans JG, Sitlani CM, Siscovick DS, King IB, Sotoodehnia N (2022). Circulating ceramides and sphingomyelins and the risk of incident cardiovascular disease among people with diabetes: the strong heart study. Cardiovasc Diabetol.

[13] Chen Y, Jia H, Qian X, Wang J, Yu M, Gong Q, An Y, Li H, Li S, Shi N (2022). Circulating palmitoyl sphingomyelin is associated with cardiovascular disease in individuals with type 2 diabetes: findings from the China Da Qing Diabetes Study. Diabetes Care.

[14] An Y, Zhang P, Wang J, Gong Q, Gregg EW, Yang W, Li H, Zhang B, Shuai Y, Chen Y (2015). Cardiovascular and all-cause mortality over a 23-year period among Chinese with newly diagnosed diabetes in the Da Qing IGT and Diabetes Study. Diabetes Care.

[15] Diabetes mellitus. Report of a WHO Study Group. World Health Organ Tech Rep Ser 1985, 727:1–113. 3934850

[16] Gong Q, Zhang P, Wang J, Ma J, An Y, Chen Y, Zhang B, Feng X, Li H, Chen X (2019). Morbidity and mortality after lifestyle intervention for people with impaired glucose tolerance: 30-year results of the Da Qing Diabetes Prevention Outcome Study. Lancet Diabetes Endocrinol.

[17] Chen Y, Zhang P, Wang J, Gong Q, An Y, Qian X, Zhang B, Li H, Gregg EW, Bennett PH, Li G (2021). Associations of progression to diabetes and regression to normal glucose tolerance with development of cardiovascular and microvascular disease among people with impaired glucose tolerance: a secondary analysis of the 30 year Da Qing Diabetes Prevention Outcome Study. Diabetologia.

[18] Lidgard B, Bansal N, Zelnick LR, Hoofnagle AN, Fretts AM, Longstreth WT, Shlipak MG, Siscovick DS, Umans JG, Lemaitre RN (2023). Evaluation of plasma sphingolipids as mediators of the relationship between kidney disease and cardiovascular events. EBioMedicine.

[19] Matthews DR, Hosker JP, Rudenski AS, Naylor BA, Treacher DF, Turner RC (1985). Homeostasis model assessment: insulin resistance and beta-cell function from fasting plasma glucose and insulin concentrations in man. Diabetologia.

[20] Sauerbrei W, Meier-Hirmer C, Benner A, Royston P (2006). Multivariable regression model building by using fractional polynomials: description of SAS, STATA and R programs. Comput Stat Data Anal.

[21] Sainani KL (2015). Dealing with missing data. PM R.

[22] Valsecchi M, Mauri L, Casellato R, Prioni S, Loberto N, Prinetti A, Chigorno V, Sonnino S (2007). Ceramide and sphingomyelin species of fibroblasts and neurons in culture. J Lipid Res.

[23] Jiang XC, Paultre F, Pearson TA, Reed RG, Francis CK, Lin M, Berglund L, Tall AR (2000). Plasma sphingomyelin level as a risk factor for coronary artery disease. Arterioscler Thromb Vasc Biol.

[24] Schlitt A, Blankenberg S, Yan D, von Gizycki H, Buerke M, Werdan K, Bickel C, Lackner K, Meyer J, Rupprecht H (2006). Further evaluation of plasma sphingomyelin levels as a risk factor for coronary artery disease. Nutr Metab.

[25] Bae M, Bandaru VV, Patel N, Haughey NJ (2014). Ceramide metabolism analysis in a model of binge drinking reveals both neuroprotective and toxic effects of ethanol. J Neurochem.

[26] Mantovani A, Lunardi G, Bonapace S, Dugo C, Altomari A, Molon G, Conti A, Bovo C, Laaksonen R, Byrne CD (2021). Association between increased plasma ceramides and chronic kidney disease in patients with and without ischemic heart disease. Diabetes Metab.

[27] Lidgard B, Hoofnagle AN, Zelnick LR, de Boer IH, Fretts AM, Kestenbaum BR, Lemaitre RN, Robinson-Cohen C, Bansal N (2023). High-density lipoprotein lipidomics in chronic kidney disease. Clin Chem.

[28] Alshehry ZH, Mundra PA, Barlow CK, Mellett NA, Wong G, McConville MJ, Simes J, Tonkin AM, Sullivan DR, Barnes EH (2016). Plasma lipidomic profiles improve on traditional risk factors for the prediction of cardiovascular events in type 2 diabetes mellitus. Circulation.

[29] Ziegler D, Strom A, Strassburger K, Knebel B, Bonhof GJ, Kotzka J, Szendroedi J, Roden M (2021). German Diabetes Study g: Association of cardiac autonomic dysfunction with higher levels of plasma lipid metabolites in recent-onset type 2 diabetes. Diabetologia.

[30] Chiu HC, Kovacs A, Ford DA, Hsu FF, Garcia R, Herrero P, Saffitz JE, Schaffer JE (2001). A novel mouse model of lipotoxic cardiomyopathy. J Clin Invest.

[31] Park TS, Hu Y, Noh HL, Drosatos K, Okajima K, Buchanan J, Tuinei J, Homma S, Jiang XC, Abel ED (2008). Ceramide is a cardiotoxin in lipotoxic cardiomyopathy. J Lipid Res.

[32] Lemaitre RN, Yu C, Hoofnagle A, Hari N, Jensen PN, Fretts AM, Umans JG, Howard BV, Sitlani CM, Siscovick DS (2018). Circulating sphingolipids, insulin, HOMA-IR, and HOMA-B: The Strong Heart Family Study. Diabetes.

[33] Li Z, Zhang H, Liu J, Liang CP, Li Y, Li Y, Teitelman G, Beyer T, Bui HH, Peake DA (2011). Reducing plasma membrane sphingomyelin increases insulin sensitivity. Mol Cell Biol.

[34] Bermudez B, Ortega-Gomez A, Varela LM, Villar J, Abia R, Muriana FJ, Lopez S (2014). Clustering effects on postprandial insulin secretion and sensitivity in response to meals with different fatty acid compositions. Food Funct.

[35] Peng G, Li L, Liu Y, Pu J, Zhang S, Yu J, Zhao J, Liu P (2011). Oleate blocks palmitate-induced abnormal lipid distribution, endoplasmic reticulum expansion and stress, and insulin resistance in skeletal muscle. Endocrinology.

[36] Depner CM, Traber MG, Bobe G, Kensicki E, Bohren KM, Milne G, Jump DB (2013). A metabolomic analysis of omega-3 fatty acid-mediated attenuation of western diet-induced nonalcoholic steatohepatitis in LDLR-/- mice. PLoS ONE.

