# Supplementary material for: Circulating palmitoyl sphingomyelin levels predict the 10-year increased risk of cardiovascular disease death in Chinese adults: findings from the Da Qing Diabetes Study
Source: Cardiovasc Diabetol. 2024 Jan 20;23:37. doi: 10.1186/s12933-023-02116-8 (PMC10800040; doi:10.1186/s12933-023-02116-8)
Supplement: Supplementary file 1 — Additional file 1: Fig. S1. Study flowchart. IGT, impaired glucose tolerance; NGT, normal glucose tolerance; PSM, palmitoyl sphingomyelin; CVD, cardiovascular disease. Fig. S2. Fractional polynomial regression. a) All-cause death, b) CVD death, and c) non-CVD death in all participants; d) all-cause death, e) CVD death, and f) non-CVD death in individuals with diabetes; g) all-cause death, h) CVD death, and i) non-CVD death in individuals without diabetes. PSM, palmitoyl sphingomyelin; CVD, cardiovascular diseases. HR calculated after adjustment for age, sex, smoking status, SBP, HbA1c, LDL-c level, creatinine level, prevalent CVD, and the use of statins and alcohol. [file 12933_2023_2116_MOESM1_ESM.docx]

**Additional materials**

**Fig. S1 Study flowchart**. IGT, impaired glucose tolerance; NGT, normal glucose tolerance; PSM, palmitoyl sphingomyelin; CVD, cardiovascular disease.

**Fig. S2. Fractional polynomial regression.** a) All-cause death, b) CVD death, and c) non-CVD death in all participants; d) all-cause death, e) CVD death, and f) non-CVD death in individuals with diabetes; g) all-cause death, h) CVD death, and i) non-CVD death in individuals without diabetes. PSM, palmitoyl sphingomyelin; CVD, cardiovascular diseases. HR calculated after adjustment for age, sex, smoking status, SBP, HbA_1c_ level, LDL-c level, creatinine level, prevalent CVD, and the use of statins and alcohol.
